# When you or a loved one becomes the statistic

**DOI:** 10.51866/mol.613

**Published:** 2024-03-04

**Authors:** Mohamed-Syarif Mohamed-Yassin

**Affiliations:** 1 MBBS, FRACGP, DipPallMed(Clin.), Department of Primary Care Medicine, Universiti Teknologi MARA, Sungai Buloh Campus, Sungai Buloh, Selangor, Malaysia. Email: syarif8258@uitm.edu.my

**Keywords:** Anticoagulant, Atrial fibrillation, Family physicians, Intracranial haemorrhage, Treatment

My father passed away from intracranial bleeding secondary to the anticoagulant that he was taking for atrial fibrillation.^1^ While trying to prevent an ischaemic stroke, he succumbed to death from intracranial bleeding.

Looking at the prescribing information, the anticoagulant he was on has slightly lower incidences of intracranial haemorrhage, haemorrhagic stroke, or fatal bleeding compared to the more traditional anticoagulant warfarin.^2^ The thing about these statistics however, is that I believe most of us would never feel that the "one" in a hundred thousand for example, would ever be one of us or our patient, let alone a close family member.

As a family physician, I offer treatment options to my patients on a regular basis. Depending on my patient's presumed level of health literacy, sometimes, I will include simple statistics on the benefits and side effects of a specific medication. From my recent reading, I realized at least two important ways that I as a doctor may influence my patients’ decision. First, doctors continue to be the most trusted profession in Malaysia and globally.^3^

Second, the way we frame our medical advice can strongly affect the choices our patients make.^4,5^ Observe the following statements:

*“You have a forty percent chance of improvement with this treatment.”*

*“There is a sixty percent chance of failure and death with this treatment.”*

Though clinically equivalent, patients are more likely to accept the recommendation of the first, positively-framed statement, compared to the second, negatively-framed statement.^4,5^

Now, look at the following statements:

*“1 in 1000 patients who take this medication will experience a major bleeding episode. ”*

*“0.1% of patients who take this medication will experience a major bleeding episode. ”*

Compared to percentages, using absolute numbers elicit stronger responses in patients as it is easier for us to imagine a crowd of real people.^4,5^

How will awareness of these change my approach to discussing medical treatments with my patients? Taking into consideration of the ever-present time constraints for consultations, I will endeavour to present the pros and cons of each option in simple language, using absolute numbers, while being aware of the positive or negative framing that I use. I will then let my patients discuss with someone they trust or seek a second opinion if they wish. If they request for my opinion, though not easy, I will try to provide it as unbiasedly as possible. I will try my best to refrain from judging them if they choose the option that I feel is less favourable. In the end, it is their life and livelihood, and if they have listened and understood my explanations, I'll support their decision.
